# Precision-cut liver slices in culture as a tool to assess the physiological involvement of Kupffer cells in hepatic metabolism

**DOI:** 10.1186/1476-5926-2-S1-S45

**Published:** 2004-01-14

**Authors:** Audrey M Neyrinck, Cristina Gomez, Nathalie M Delzenne

**Affiliations:** 1 Unité de Pharmacocinétique, Métabolisme, Nutrition et Toxicologie, Département des Sciences Pharmaceutiques, Université Catholique de Louvain, PMNT-UCL 73 avenue Mounier, B-1200 Brussels, Belgium

## Introduction

Hepatic macrophages have the capacity to secrete a tremendous array of molecules, which can be divided into 3 categories é cytokines (TNF-alpha), lipid mediators (prostaglandins PGE_2_) and reactive intermediates (NOé) é in response to stimulus, such as lipopolysaccharides (LPS) [1,2]. Such mediators are capable to modulate both the metabolism and the integrity of hepatocytes in vitro [2]. The physiological role of Kupffer cell in hepatic metabolism regulation has been approached in the present study by using the original in vitro model of precision-cut liver slices (PCLS) in culture; this model allows preserving the liver lobule architecture, by maintaining namely cell diversity in physiological proportion and cell-cell interactions [3]. First, we established whether non-parenchymal cells are still viable in rat PCLS and are able to respond to LPS *in vitro*; TNF-alpha, PGE_2_, NOx (reflecting NOé release) were measured in the incubation medium of PCLS from rats previously treated with GdCl_3 _é a specific inhibitor of Kupffer cell phagocytosis [4] é or NaCl as a control- in order to evaluate the contribution of Kupffer cell in mediator release. Moreover, by using the same model, we have investigated the role of Kupffer cell in the regulation of lipid synthesis in PCLS, in order to approach the biochemical mechanism explaining our last results, which indicate that the inhibition of Kupffer cell by GdCl_3 _leads to triglycerides accumulation in liver tissue [5].

## Methods

### Materials

Male Wistar rats weighing 240é280 g were used for the preparation of PCLS or for isolation of hepatocytes. Most chemicals of purest grade available were purchased from Sigma (Filter Service, Belgium), Roche Diagnostics Belgium or Invitrogené (Belgium). [1-^14^C]-acetic acid (specific activity 60 mCi/mmol) was obtained from Amersham Pharmacia Biotech Europe (Buckinghamshire, United Kingdom).

### Study of mediator secretion by PCLS in culture

PCLS were prepared from treated with GdCl_3 _(10 mg/kg i.v) (Gd+) or NaCl 0.9% (Gd-) 24 h before liver removal according to a procedure previously described [6] and were incubated in William's E medium, supplemented with penicillin (100 IU/ml), streptomycin (100 micrograms/ml), glutamine (2 mM), insulin (100 nM) and bovine serum albumin 0.1 %) containing LPS at 0 é 0.1 é 10 micrograms/ml. Medium was frozen after 2 h and 20 h of incubation for further analysis. PGE_2 _and TNF-alpha concentration were measured in frozen aliquots incubation medium with immunoassay kits (PGE_2 _Immunoassay, DE0100 and Quantikine rat TNF-alpha immunoassay, RTA00 from R&D Systems) whereas NOx (NO_2_^- ^+ NO_3_^-^) concentration was measured by the Griess reaction [7]. ATP content of PCLS was greater than 8 nmol/mg protein in all experiments.

### Study of lipid synthesis by PCLS

PCLS were prepared from treated with GdCl_3 _(10 mg/kg i.v) (Gd+) or NaCl 0.9% (Gd-) 48 h before liver removal according to a procedure previously described [6]. PCLS were incubated as described above. Blood was collected from vena cava for serum PGE_2 _measurement. After 2 h of preincubation, medium was frozen for further analysis and PCLS were transferred into fresh medium containing 2 mM [^14^C]-acetate (0.2 mCi/mmol); after 3 h of incubation, PCLS were sonicated in 0.5 ml NaCl 0.05 M before lipid extraction and separation by thin-layer chromatography with hexane/ether/acetic acid (80:20:1) [8,9]. Spots corresponding to triglycerides, phospholipids and cholesterol were scrapped from the plate and counted in 10 ml scintillation fluid (Ultima Gold) in a beta counter.

### Study of lipid synthesis by isolated hepatocytes in suspension

The hepatocytes were isolated from fed animals [10], through perfusion with a buffer containing Liberaseé (35 micrograms/ml). Cells were incubated in the presence PGE_2 _(Cayman Chemicals) dissolved in DMSO at 10 micromolar and 2 mM [^14^C]-acetate (0.2 mCi/mmol) in the same medium described above. After 15 min of incubation, hepatocytes were pelleted and sonicated in 0.5 ml NaCl 0.05 M before lipid extraction according to Folch [8]. The chloroform phase was counted in 10 ml scintillation fluid (Ultima Gold) in a beta counter.

### Statistical analysis

Values are presented as means é S.E.M. In the study of mediator secretion by PCLS, statistical analysis was performed by two-way ANOVA with the Tukey's post hoc test (with SPSSé statistical software). Other data were analysed by Student-*t *test. Statistical significance was set at p &lt; 0.05.

## Results

### Validation of the PCLS model to assess the functionality of Kupffer cells

**Figure 1:**
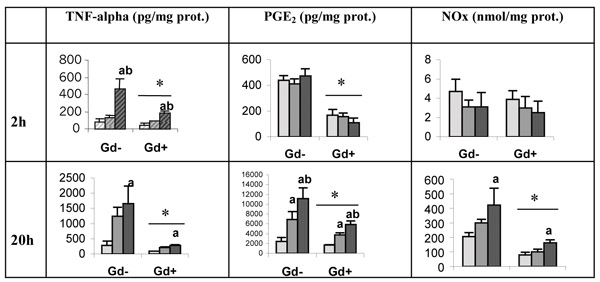
Effect of Kupffer cell inhibition on mediator secretion (TNF-alpha, PGE_2 _and NOx) by PCLS, obtained from rats pretreated with GdCl_3 _(Gd+) or NaCl (Gd-) 24 h before experiment; PCLS were incubated during 2 h and 20 h in the presence of LPS (from left to right, each group of three bars correspond to: 0 micrograms/ml, 0.1 micrograms/ml, 10 micrograms/ml). Values are means é S.E.M (n é 3; *p &lt; 0.05 Gd+ vs Gd-, two-way ANOVA; ^a ^p &lt; 0.05 LPS 10 micrograms/ml vs LPS 0 micrograms/ml and ^b ^p &lt; 0.05 LPS 10 micrograms/ml vs LPS 0.1 micrograms/ml, two-way ANOVA followed by Tukey's post hoc test).

Unstimulated-PCLS in culture release in the medium significant amount of TNF-alpha, PGE_2 _and NOx (Figure 1) which level increases with the time of incubation. TNF-alpha concentration increases significantly in the presence of LPS; the effect is dependent on the dose of LPS and is already present after 2 h of incubation. The extent of both PGE_2 _and NOx production, is also dependent on LPS concentration; the increase in both parameters appears significantly only after 20 h of incubation. The administration of GdCl_3 _1 day before PCLS preparation strongly reduces the basal production of the 3 mediators measured as well as upon stimulus by LPS.

### Role of Kupffer cell in the physiological regulation of lipid synthesis in the liver tissue

PCLS obtained from rats treated with GdCl_3_, 2 days before experiment, are characterised by a higher incorporation of [^14^C]-acetate into lipids (table 1), on one hand, and by a lower release of PGE_2 _in the preincubation medium, on the other hand (PGE_2 _concentration in the medium after 2 h: 5.4 é 0.8 nM and 0.9 é 0.1 nM for Gd- and Gd+ rats respectively; *p &lt; 0.05, Student t-test). Moreover, PGE_2_, measured in the serum obtained from vena cava, was much lower in animals treated with GdCl_3 _2 days before blood sampling (&lt; 1 nM) as compared to control rats (26.7 é 12.1 nM). The role of PGE_2 _in the short-term control of lipid synthesis was assessed by incubation of isolated hepatocytes in suspension: the addition of PGE_2 _(10 micromolar) to the culture medium rapidly decreases the incorporation of [^14^C]-acetate into total lipids in isolated hepatocytes after 15 minutes of incubation, total lipids é expressed in nmol acetate equivalent/mg protein é reached 1.3 é 0.2 and only 0.87 é 0.1 in control conditions (DMSO) and after addition of PGE_2 _10 micromolar, respectively (*p &lt; 0.05, Student t-test).

**Table 1:** Effect of Kupffer cell inhibition by GdCl_3 _on lipid synthesis by PCLS

Acetate equivalent incorporated into lipids (nmol/mg prot.)	**Gd-**	**Gd+**
- phospholipids	3.7 é 0.1	6.1 é 0.6*
- triglycerides	2.4 é 0.6	4.0 é 0.8*
- cholesterol	0.8 é 0.2	2.0 é 0.2*

PCLS, prepared from 24 h-fasted rats pretreated with GdCl_3 _(Gd+) or NaCl (Gd-) 2 days before the experiment, were incubated in medium containing 2 mM [^14^C]-acetate for 3 h. Values are means é S.E.M (n é 4) (*p &lt; 0.05, Student t-test).

## Discussion

Our study demonstrates that GdCl_3_, a specific inhibitor of Kupffer cell phagocytosis [4], decreases the rate of LPS-induced TNF-alpha, PGE_2 _and NOx production by PCLS; those 3 molecules are known as typical mediators produced by macrophages (and namely by Kupffer cells in culture) upon stimulation by LPS (cytokines, lipid mediators and reactive intermediates) [10]. Moreover, we have shown that PCLS are able, independently of any stimulus (absence of LPS or any toxic agents) to produce TNF-alpha, NOx and PGE_2_. In this case also, the release of mediators is decreased by previous GdCl_3 _treatment. Therefore, we conclude that PCLS from GdCl_3_-treated rats é compared to PCLS obtained from control rats é is a convenient in vitro system to study the complex Kupffer cell-hepatocyte interactions retained inside the liver tissue, in basal conditions or after an inflammatory stimulus. PGE_2 _merits special attention: when PCLS are prepared from Gd+ animals, PGE_2 _release is strongly depressed (mainly during short time incubation) suggesting that Kupffer cells are important producers of PGE_2 _by the liver. Since GdCl_3 _treatment leads to a strong depression of PGE_2 _concentration in the serum, we may propose that Kupffer cells inside the liver constitute an important source of circulating PGE_2_, known to exert pleiotropic effects outside the liver (control of lipolysis, platelet aggregation, gastric acid secretion, immunoregulation, neurotransmitter release, contraction-relaxation of smooth muscle) [11]. On the other hand, we show here that PGE_2 _release by Kupffer cells, not only plays a role in the systemic availability of this prostaglandin, but is also able to exert paracrine effect on hepatocyte, with relevant metabolic consequences in the liver tissue: we had previously shown that, *in vivo*, the accumulation of lipids in the liver tissue after GdCl_3 _administration, modifies the histological image of the liver, revealing steatosis [6]. We have demonstrated in the results presented here that the inhibition of Kupffer cells by GdCl_3 _leads to a higher cholesterol, triglycerides and phospholipids synthesis by PCLS, a phenomenon related to a lower PGE_2 _release. Since PGE_2 _alone added in the culture medium of isolated hepatocytes decreases lipid synthesis, we may postulate that the lower PGE_2 _secretion after GdCl_3 _treatment is, at least partly, responsible for a higher lipid synthesis inside the liver tissue. This is in favour of a role of Kupffer cell-released PGE_2 _in the physiological control of lipid synthesis in hepatocytes. By which mechanism PGE_2 _could control lipid synthesis in the liver tissue? The phosphorylation rate of hepatic enzymes involved in the lipogenesis or cholesterogenesis pathways (acetyl-CoA synthetase, HMG-CoA-reductase) could be modulated by PGE_2 _which is released at concentration sufficient to alter the phosphorylation of specific proteins inside the hepatocytes [12]. The use of indomethacin, known to inhibit prostaglandin production, in the incubation medium of PCLS could help to elucidate the role of PGE_2_released by Kupffer cell in the regulation of hepatic metabolism.

## References

[B1] Decker K (1990). Biologically active products of stimulated liver macrophages (Kupffer cells). Eur J Biochem.

[B2] Laskin DL, Pendino KJ (1995). Macrophages and inflammatory mediators in tissue injury. Annu Rev Pharmacol Toxicol.

[B3] C Lerche-Langrand, Toutain HJ (2000). Precision-cut liver slices: characteristics and use for in vitro pharmaco-toxicology. Toxicology.

[B4] Hardonk MJ, Dijkhuis FWJ, Hulstaert CE, Koudstaal J (1992). Heterogeneity of rat liver and spleen macrophages in gadolinium chloride-induced elimination and repopulation. J Leukoc Biol.

[B5] Neyrinck AM, Taper HS, Delzenne NM, Wisse E, Knook DL, de Zanger R, Fraser R (2001). Gadolinium chloride does not block the hypertriglyceridemia induced by LPS administration to rats. Cells of the Hepatic sinusoid.

[B6] Neyrinck AM, Taper HS, Gevers V, Declerck B, Delzenne NM (2002). Inhibition of Kupffer cell activity induces hepatic triglyceride synthesis in fasted rats, independent of lipopolysaccharide challenge. J Hepatol.

[B7] Bishop-Bailey D, Larkin SW, Warner TD, Chen G, Mitchell JA (1997). Characterization of the induction of nitric oxide synthase and cyclo-oxygenase in rat aorta in organ culture. Br J Pharmacol.

[B8] Folch J, Lee M, Sloane-Standley G (1957). A simple method for the isolation and purification of total lipids from animal tissues. J Biol Chem.

[B9] Liao W, Florén CH (1994). Upregulation of low density lipoprotein receptor activity by TNF, a process of TNF-induced lipid synthesis and secretion. Lipids.

[B10] Roland CR, Naziruddin B, Mohanakumar T, Flye MW (1996). Gadolinium chloride inhibits Kupffer cell nitric oxide synthase (iNOS) induction. J Leukoc Biol.

[B11] Coleman RA, Smith WL, Narumiya S (1994). International union of pharmacology classification of prostanoid receptors: properties, distribution and structure of the receptors and their subtypes. Pharmacological Reviews.

[B12] Casteleijn E, Kuiper J, Van Rooij HC, Koster JF, Van Berckel TJ (1988). Conditioned media of Kupffer and endothelial cells influence protein phosphorylation in parenchymal liver cells. Involvement of prostaglandins. Biochem J.

